# Herpes zoster frequency and severity in immunocompromised pediatric populations: a structured narrative review

**DOI:** 10.3389/fped.2026.1900439

**Published:** 2026-08-28

**Authors:** Chris Simpson, Emma Viscidi, Adva Gadoth

**Affiliations:** 1 Epidemiologic Research & Methods LLC, Atlanta, GA, United States; 2 Moderna Inc., Cambridge, MA, United States

**Keywords:** autoimmune, epidemiology, herpes zoster (HZ), immunocompromised, pediatrics, varicella and herpes zoster, varicella vaccination

## Abstract

**Background:**

While herpes zoster (HZ) is known to disproportionately affect people with immunocompromising (IC) conditions, reviews of the frequency and severity of HZ in these conditions have so far been limited to adults.

**Methods:**

For this narrative review, we conducted a literature search to identify studies describing HZ frequency in pediatric cohorts with IC conditions including hematopoietic cell transplant, cancer, solid organ transplant, human immunodeficiency virus (HIV), or autoimmune disease commonly treated with immunosuppressants. We also extracted information on HZ complications, when available.

**Results:**

Forty-one articles published between 1997 and 2024 from 22 countries were identified. Thirty-four reported risk (cumulative incidence) and 21 reported incidence rates of HZ. The frequency of HZ was elevated in all IC conditions studied compared to that in the general pediatric population. Risk of HZ was particularly high among children receiving HCT (median cumulative incidence: 18%; median follow-up: two years) and children with systemic lupus erythematosus (SLE) (median cumulative incidence: 26%; median follow-up: six years). Children with inflammatory bowel disease had HZ incidence rates approximately three times that of controls. Across all IC conditions, postherpetic neuralgia was less common and resolved faster than in immunocompromised adults, but acute complications were no less common. Varicella vaccination was associated with lower HZ risk in all studies that reported on this intervention.

**Conclusions:**

Children with IC and autoimmune conditions, particularly HCT and SLE, develop HZ more frequently and are more likely to experience HZ complications than other children. Varicella vaccination appears to reduce these risks.

## Highlights

- Herpes zoster (HZ) occurs more frequently and involves greater complications in children with immunocompromising conditions.
- HZ risk is especially high in hematopoietic cell transplant recipients.
- Among autoimmune conditions studied in children, systemic lupus erythematosus confers the highest HZ risk.

## Background

Herpes zoster (HZ) is a reactivation of latent varicella zoster virus (VZV) characterized by a painful and/or itchy vesicular rash. HZ is less common in children than in adults, with pediatric incidence rates estimated at <1 to 2 cases per 1,000 person-years (PY) (1–3) and adult rates ranging from 4 to 5 cases per 1,000 PY (4–6).

The greatest contributor to the health burden of HZ—postherpetic neuralgia (PHN)—is far less likely to develop in children than adults. Risk of PHN has been estimated at 0.11% below age 15 (7), <1% in ages 0–9, and <1% in ages 10–19 (8). Studies of adults ages 18+ or 20+ years report PHN risk ranging from 6% to 26% (9).

Immunocompromising (IC) conditions predispose both children and adults to HZ by increasing the risk of viral infection and reactivation. A study of ∼6.4 million children in a US managed care organization found that children classified as immunocompromised had 5.7 times the rate of HZ compared with children not classified as such (10). Additionally, a broad literature addresses the frequency and outcomes of opportunistic infections among cohorts with specific IC conditions, but no work to our knowledge has yet summarized findings related to HZ risk and manifestations across pediatric IC conditions.

A 2020 literature review synthesized literature on HZ frequency and complications among US *adults* with four IC conditions: hematopoietic cell transplant (HCT), cancer, solid organ transplant (SOT), and human immunodeficiency virus (HIV) treated with highly active antiretroviral therapy (HAART) (11). The current review characterizes HZ disease burden among *pediatric* populations globally across these same IC conditions and additionally includes children with autoimmune diseases, in whom immunosuppressive therapies are commonly used.

## Methods

A search of peer-reviewed literature published between 1 January 1993 and 15 September 2024 was conducted in PubMed. The search terms used by an earlier review focused on HZ in adults were adapted to identify pediatric subjects, include autoimmune diseases, and not exclude any countries (11). Medical subject headings and keywords related to age were used to improve the specificity of search results for pediatric age groups while limiting loss of sensitivity. Full search details are described in Supplementary Materials.

Titles and abstracts of identified articles were screened by two reviewers, utilizing pre-specified inclusion and exclusion criteria, for their potential to report on the frequency of HZ in pediatric patients with an IC condition of interest (HCT, cancer, SOT, HIV on HAART, or any autoimmune disease) or to reference articles that did. Frequency was considered to include either: 1) cumulative incidence (henceforth, “risk”) – i.e., the proportion of all study subjects who developed HZ during follow-up, or 2) incidence rate (IR)—i.e., the number of incident HZ cases per unit of person-time at risk during follow-up. Review articles and other secondary sources were not eligible for inclusion, but they were not excluded from the PubMed search because of their potential to help identify primary sources missed by the search. More generally, the reference lists of all articles passing the initial title and abstract screening were reviewed for potentially relevant papers.

**Figure 1:**
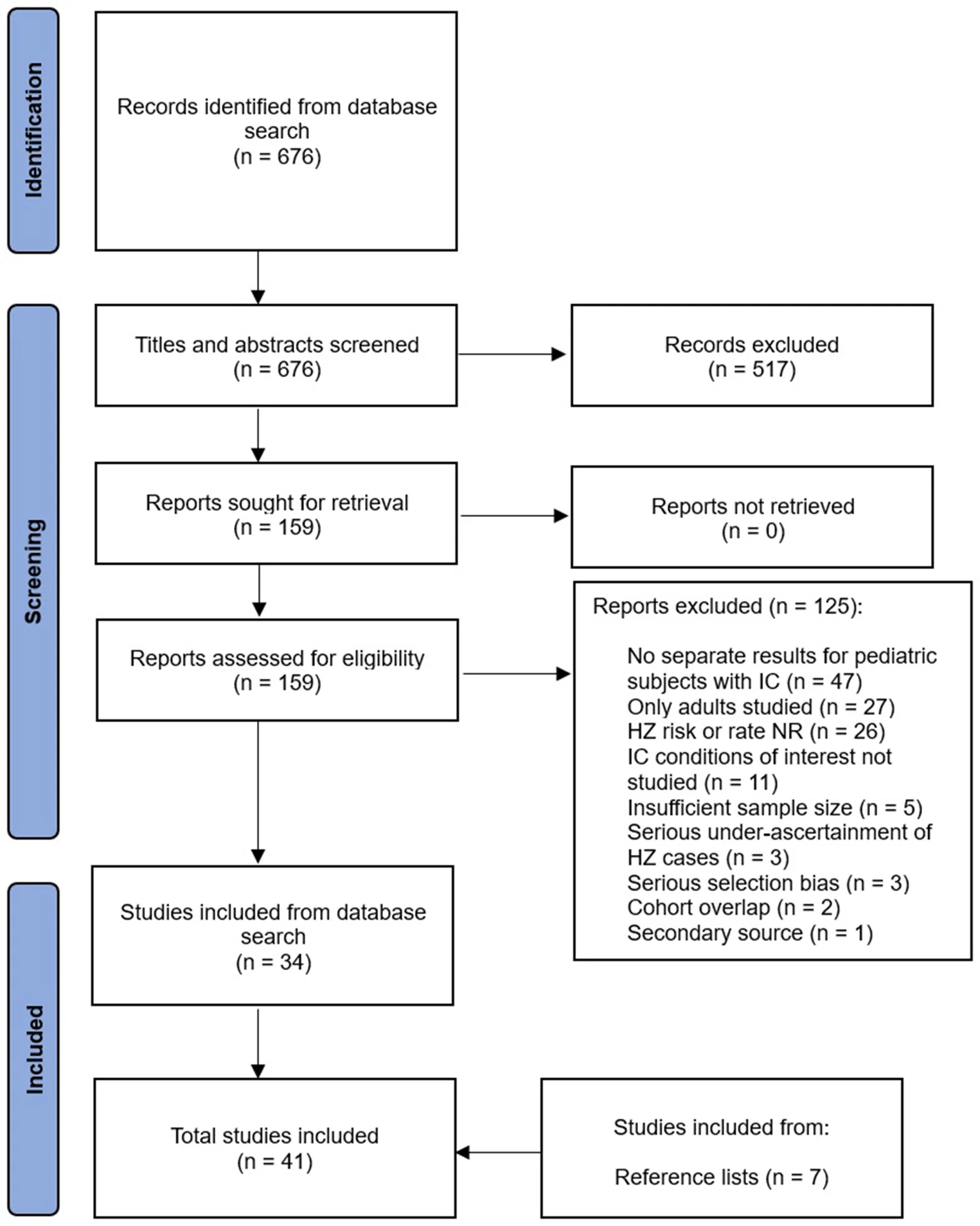
Flow diagram of study selection.

Upon full text review, if a study was found to report HZ risk or IR for at least five pediatric subjects with the same IC condition of interest (or provided sufficient information that permitted the calculation of either), was not subject to severe biases (as when a study lacked the ability to detect HZ cases not resulting in hospitalization), and did not report on the same or a similar cohort covered by another included study, then it was included (Figure 1). To prioritize more recent studies, it was decided after initial title/abstract screening to limit full text review to papers published since 2000. (This decision affected 24 papers published 1993–1999 that otherwise passed title/abstract screening; in Figure 1, these papers are counted among the 517 records excluded at title/abstract screening.) However, due to the dearth of papers (*n* = 2) regarding SOT and cancer outside the context of HCT, an exception was made for these two particular IC conditions, and papers over the entire temporal range of the search (i.e., going back to 1993) were reviewed. In the case of multiple studies describing “overlapping cohorts,” the study with the larger sample size was retained. A cohort was considered pediatric if: 1) most of the person-time at risk for HZ was among subjects under 20 years old, and 2) the oldest subject at any point during follow-up was still in his or her 20s. When information was presented graphically but not textually, the software WebPlotDigitizer 4.6 (63) was used to assist the extraction of numerical data points from figures.

The following information was extracted for all included studies:

- Primary HZ frequency outcomes
  ○ Study-wide HZ risk (i.e., cumulative incidence)
  ○ Study-wide incidence rate (IR) of HZ
- Secondary or comparative HZ frequency outcomes
  ○ HZ risk at interim follow-up lengths (e.g., 6 months, 1 year)
  ○ HZ risk and/or IR among internal study controls
  ○ HZ risk and/or IR for relevant subgroups (e.g., different pediatric age groups)
- Covariates
  ○ IC condition(s) of interest
  ○ Study setting and design
  ○ Sample size (i.e., number of individuals with IC condition of interest)
  ○ Ages of subjects (at start of follow-up, at HZ onset)
  ○ Average duration of follow-up
  ○ Use of antiviral prophylaxis
  ○ Frequency of varicella vaccination (VV) and association with primary HZ frequency outcomes
  ○ For HCT, underlying disease condition(s) and type of transplant (allogeneic vs. autologous)
  ○ Names and counts of HZ complications (including zeros)

Because immunocompromised children with HZ are often hospitalized preemptively for prophylactic treatment and monitoring, hospitalization rates in this population are likely not accurate markers of HZ disease severity and, therefore, were not included among the outcomes of interest. Studies enrolling fewer than five total participants were excluded because they are unlikely to yield epidemiologically meaningful estimates of HZ frequency and may introduce bias through inclusion of highly selected or atypical patient populations.

## Results

The PubMed search yielded 676 articles, of which 159 underwent full-text review and 34 were included. In addition, seven papers identified through reference lists of the reviewed papers were included, for a total of 41 studies in the current review (Figure 1). Papers involving HCT (18) or autoimmune disease (15) predominated, together comprising 80% of included studies. Among autoimmune diseases, there were six relevant studies of systemic lupus erythematosus (SLE), five of inflammatory bowel disease (IBD), and four of juvenile idiopathic arthritis (JIA). Other papers involved HIV (four papers), kidney transplant recipients (two), and acute lymphoblastic leukemia (two); one paper involved all malignancies (with leukemias, lymphomas, and solid tumors reported separately). Study characteristics are presented in Table 1.

**Table 1:** Studies reporting occurrence of herpes zoster in pediatric populations by immunocompromising condition.

Author, Year	Country (study period)	Primary data source	Age	Follow-up time per subject (y)	Sample size
Inflammatory Bowel Disease (IBD)					
Côté-Daigneault et al., 2019 (26)*	Canada (1999-2015)	Administrative data	0-17	NR (likely > 5)	NR (39,366 for all ages)
Chang et al., 2018 (27)*	South Korea (2009-2013)	Administrative data	0-19	2.0	4,282
Long et al., 2013 (29)*	United States (1997-2009)	Administrative data	0-9, 10-19	NR [med: 2.8 for CD, 3.0 for UC for all study ages (0-64)]	NR [*N* = 50,932 for CD, *N* = 56,403 for UC for all study ages (0–64)]
Gupta et al., 2006 (54)	United Kingdom (1988-1997)	Medical records (EMR)	0-4, 5-14	≥ 1	NR (19,753 for all ages)
Friesen et al., 2004 (28)	United States (1998-2003)	Medical records	mean: 13.4 (range: 4-20)	1.7	111
Juvenile Idiopathic Arthritis (JIA)					
Brunelli et al., 2018 (22)	Brazil (2004-2016)	Medical records (EMR)	0-25 (mean: 14.6; SD: 5.7)	3.7	107
Nicolai et al., 2016 (23)	Italy (NR-2010)	Medical records	mean: 6.9	2.5	156
Nimmrich and Horneff, 2015 (24)	Germany (2000-2012)	Medical records (clinical trial data)	mean: 11.0	1.8	3,042
Beukelman et al., 2013 (25)	United States (2000-2005)	Administrative data	med: 10.1 (IQR: 5.9, 13.3)	1.6	8,503
Systemic Lupus Erythematosus (SLE)					
Yu et al., 2022 (16)	Taiwan (2005-2013)	Administrative data	0-17	NR for 0-17 (but based on study design, likely very similar to that for all ages: med: 8.4; IQR: 8.1, 8.7)	147
Ferreira et al., 2016 (15)^^^	Brazil (2012-2014)	Medical records	0-24 (med: 15)	1-2	852
Costa-Reis et al., 2013 (17)	United States (1991-2009)	Medical records	0-17 (IQR: 10, 14)	5.0	120
Chen et al., 2011 (18)*	Taiwan (1998-2006)	Administrative data	0-17	3 for all subjects	955
Wu et al., 2011 (19)	Taiwan (1999-2008)	Medical records	mean: 12.9 (range: 6.8-17.1)	4.9	98
Lee et al., 2006 (20)	Hong Kong (1998-2004)	Medical records	0-17 (mean: 10.9; SD: 3.6)	6.8	49
Hematopoietic Cell Transplant (HCT)					
Arici et al., 2024 (12)^^^	Turkey (1996-2020)	Medical records	med: 10.4 (range: 0.5-18.4)	med: 6.4	273
Tatebe et al., 2022 (55)	Japan (2008-2018)	Medical records	med: 4.8 (range: 0.7-14.6)	med: 2.0	53 (allogeneic: 37, autologous: 16)
Düver et al., 2020 (14)^^^	Germany (2005-2015)	Medical records	med: 9.0 (range: 0.2-22.2)	med: 3.8	107
Kang et al., 2020 (32)	South Korea (1998-2013)	Medical records	< 21 (med: 4.8)	med: 1-2	413
Ryan et al., 2019 (56)	Australia (2005-2017)	Medical records	mean: 7.2 (range: 0.2-17.0)	range: 0.44-1.08	71
Han et al., 2017 (33)	South Korea (2009-2014)	Medical records	med: 9-10 (range: 0-19)	med: 2.1	217
Choi et al., 2016 (57)	South Korea (2004-2014)	Medical records	med: 4.8 (range: 0.6-27.4)	slightly < 30 d (max: 30 d)	607
Goussetis et al., 2015 (58)	Greece (1995-2013)	Medical records	med: 11.0	med: 10	105
Blennow et al., 2014 (59)	Sweden (2000-2012)	Medical records	0-19	NR (med: 2.4, range: 8 d to 12.8 y for all ages)	NR (802 for all ages)
Vermont et al., 2014 (60)	Netherlands (2002-2007)	Medical records	med: 8.5 (range: 0.2-19.0)	med: < 1	163
Aytac et al., 2011 (34)	Turkey (2000-2006)	Medical records	med: 7 (range: 0.5-20.0)	NR	125
Sørensen et al., 2011 (31)	Denmark (1992-2007)	Medical records	1-14	2.5	30
Onozawa et al., 2009 (37)	Japan (2005-2007)	Medical records	0-9, 10-19	NR (med: 0.9, range: 3 d to 3.2 y for all ages)	NR (418 for all ages)
Vandenbosch et al., 2008 (61)	Canada (1996-2003)	Medical records	med: 9 (range: 0.1-21)	max: 3	114
Berman et al., 2006 (36)	United States (1995-2002)	Medical records	med: 7-8 (allogeneic: 9, autologous: 5)	max: 8	310 (allogeneic: 201, autologous: 109)
Barker et al., 2005 (30)	United States (1994-2002)	Medical records	med:8	0.95	136
Leung et al., 2000 (35)	Hong Kong (1991-1998)	Medical records	med: 9.2 (range: 1.3-21.1)	med: 3.0 for cases, about 1-2 for non-cases	109 (allogeneic: 96, autologous: 13)
Maltezou et al., 2000 (62)	United States (1992-1996)	Medical records	med: 10 (range: 1-22)	med: 0.6	96
Cancer					
Lin et al., 2016 (39)*	Taiwan (2000-2008)	Administrative data	0-18 0-18 0-18 0-18	3.38 3.23 3.58 3.43	4,432 any cancer 1,450 leukemia 532 lymphoma 2,450 solid turmor
Sørensen et al., 2011 (31)	Denmark (1992-2007)	Medical records	med: 5 (range: 1-14)	2.9	226 (acute lymphoblastic leukemia)
Gershon et al., 1996 (38)	United States (1980-1992)	Medical records (clinical trial data)	mean: 5.6	4.0	511 (acute lymphoblastic leukemia)
Solid Organ Transplant (Kidney)					
Chavers et al., 1997 (40)	United States (1985-1993)	Medical records	0-17	4.6	164
Broyer et al., 1997 (13)^^^	France (1973-1992)	Medical records	NR [“children and adolescents”; mean age at HZ: 15.6 (range: 5-22); mean time from transplant to HZ: 3.3 yr (range: 0.2-11)]	Entire cohort: likely 5-6 (range: 0.3-22) with VV: 4.8	704 212
HIV					
Nesheim et al., 2013 (41)	United States (2005-2006)	Medical records	mean: 9.2 (range: 0.05, 24.8)	max: 0.45	524
Alarcón et al., 2012 (44)	Brazil, Mexico, Argentina, Peru, and Jamaica (2002-2007)	Medical records	med: 5.0 (IQR: 3.0, 9.0)	med: 3.9 (IQR: 3.3, 4.3)	731
Wood et al., 2008 (43)	United States (2000-2006)	Medical records	NR (likely similar to the other HIV studies in this table)	med: 3.1 (IQR: 1.7, 4.9)	57
Gona et al., 2006 (42)	United States and Puerto Rico (2001-2004)	Medical records	med: 9.9 (IQR: 6, 12)	med: 3.4 (IQR: 1.4, 4.0)	2,767

Unless stated otherwise, age information is as of the start of the follow-up period. Follow-up time per subject is mean unless indicated differently.

All studies were retrospective cohort studies except: Nimmrich and Horneff (24) (open-label trial); Wu et al. (19) (prospective cohort); Ryan et al. (56) (prospective cohort); Barker et al. (30) (prospective cohort); Gershon et al. (38) (open-label trial); Alarcón et al. (44) (prospective cohort); and Gona et al. (42) (prospective cohort).

IBD, inflammatory bowel disease; NR, not reported; med, median; CD, Crohn's disease; UC, ulcerative colitis; EMR, electronic medical record; JIA, juvenile idiopathic arthritis; SD, standard deviation; IQR, interquartile range; SLE, systemic lupus erythematosus; HCT, hematopoietic cell transplant; HZ, herpes zoster; VV, varicella vaccination; HIV, human immunodeficiency virus.

* Study included a matched control group without the condition.

^ Study reported HZ risk stratified by VV status. Vaccination rates ranged from 2.8% to 31.4% across the studies reporting VV status.

The methodologic quality of the studies was deemed appropriate for the purposes of this narrative review, and was assessed on the following domains: study design, length/ completeness of follow-up, presentation of patient characteristics during follow-up (including use or discontinuation of immunosuppressive or antiviral prophylactic drugs), ascertainment of HZ, and other outcome assessment (including post-herpetic neuralgia). Because of the generally severe nature of the IC conditions of interest, patients in the included studies tended to receive close medical monitoring, which supported the completeness and accuracy of HZ case ascertainment. Most studies (83%) used medical records as the primary data source, which is the gold standard for epidemiologic studies of the frequency of medically-attended HZ. Thirty-nine studies (95%) were cohort studies (34 retrospective, 5 prospective) and two (5%) were open-label trials. Because study populations sometimes differed with respect to factors potentially associated with HZ risk – such as the treatment(s) used for management of the IC condition – additional information characterizing the patient populations of each study is presented in Supplementary Table 1.

**Figure 2:**
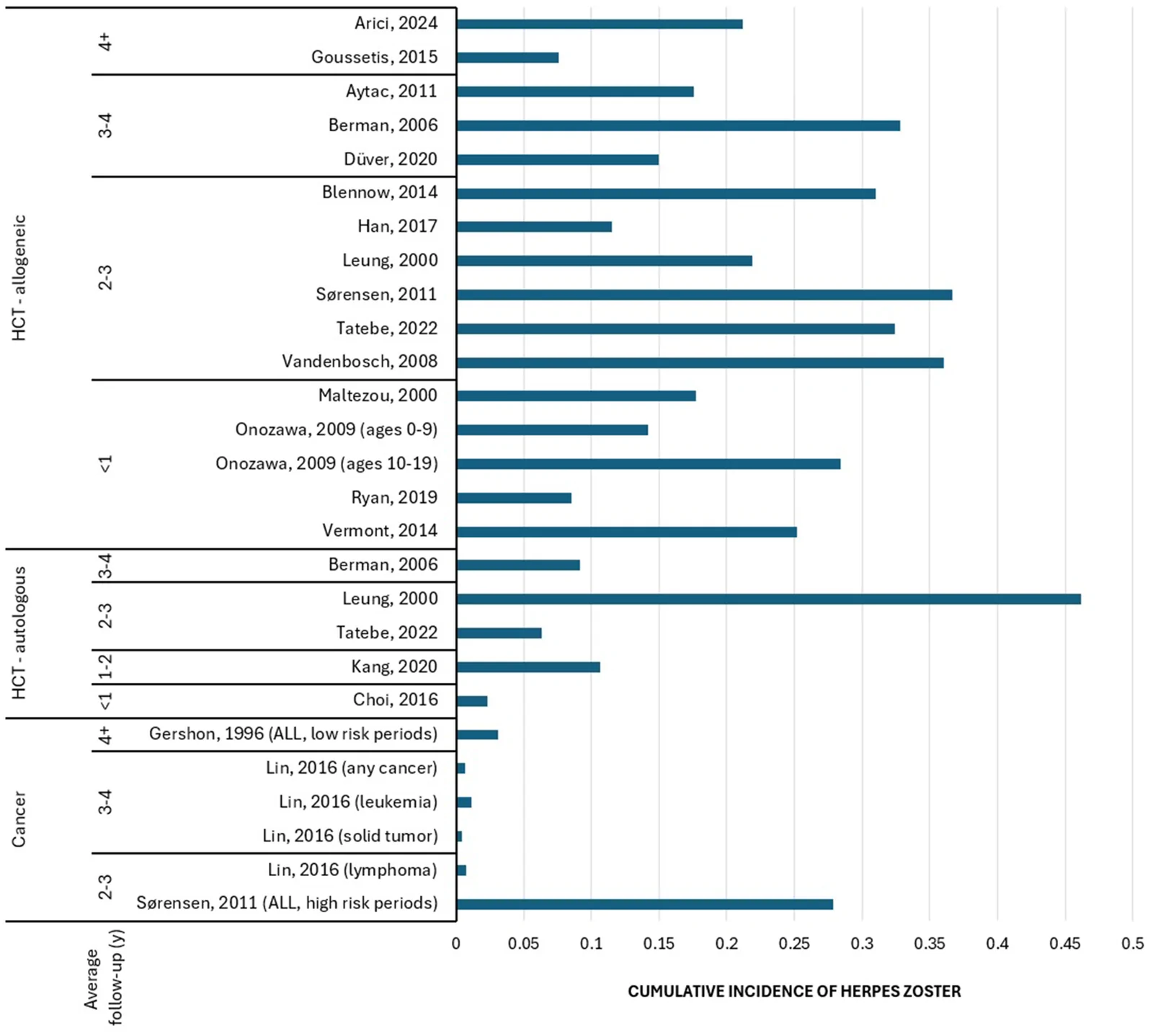
Risk (cumulative incidence) of herpes zoster in pediatric HCT and cancer patients. ALL, acute lymphoblastic leukemia; HCT, hematopoietic cell transplant. Onozawa et al. (37) reported results separately for ages 0-9 and ages 10-19. Results from Lin et al. (39) are presented separately for different types of cancer (from left to right: any cancer, leukemia, solid tumors, lymphoma).

**Figure 3:**
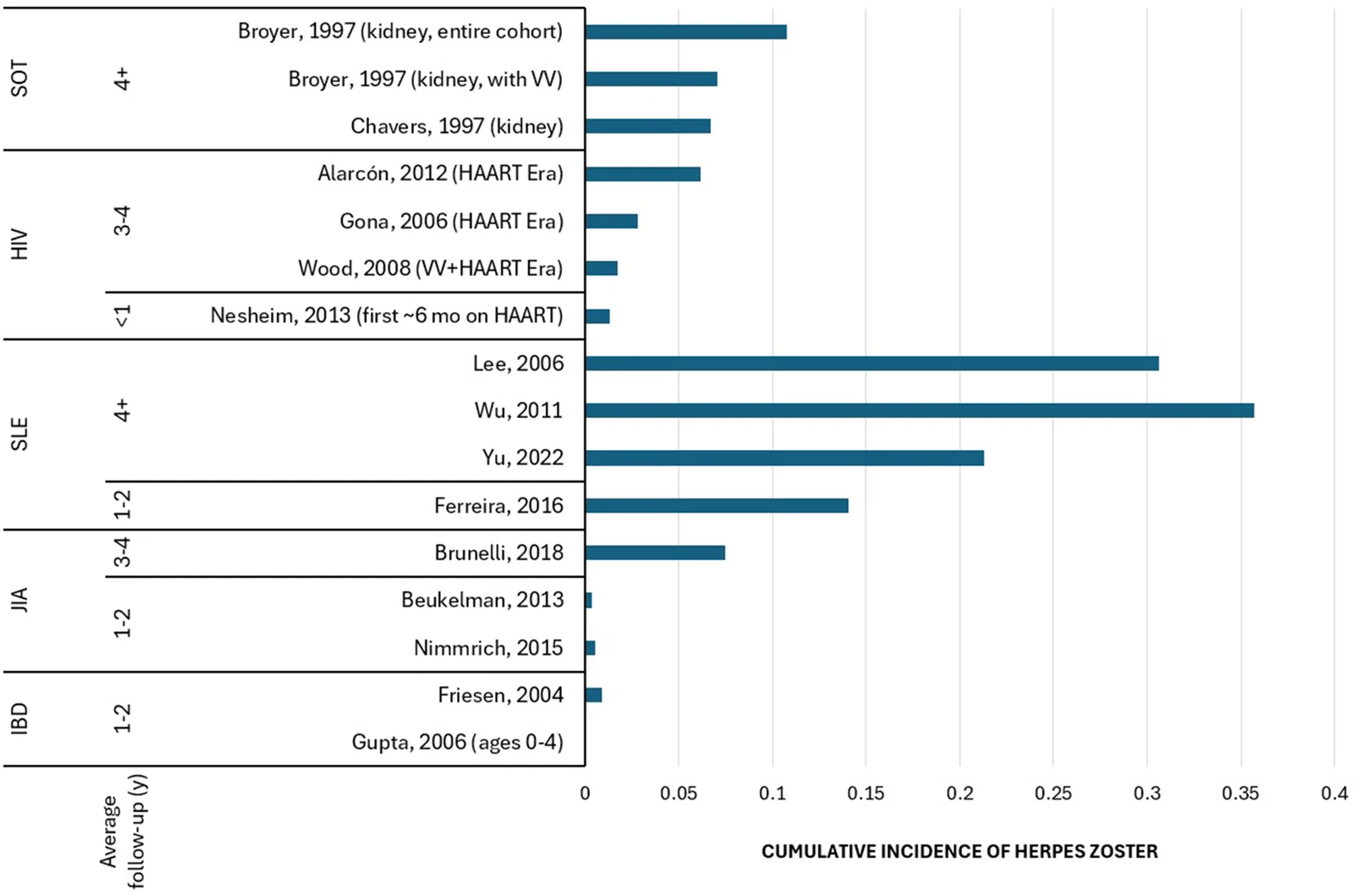
Risk (cumulative incidence) of herpes zoster in pediatric organ transplant, HIV, and autoimmune disease patients. HAART, highly active antiretroviral therapy; HIV, human immunodeficiency virus; IBD, inflammatory bowel disease; JIA, juvenile idiopathic arthritis; SLE, systemic lupus erythematosus; SOT, solid organ transplant; VV, varicella vaccination. Results from Broyer et al. (13) are presented for the entire cohort (left, *n* = 676), and for the subset with history of varicella vaccination (right, *n* = 212). The result from Gupta et al. 2006 (52) was for ages 0-4, in whom no HZ cases were observed (hence the absence of a bar).

**Figure 4:**
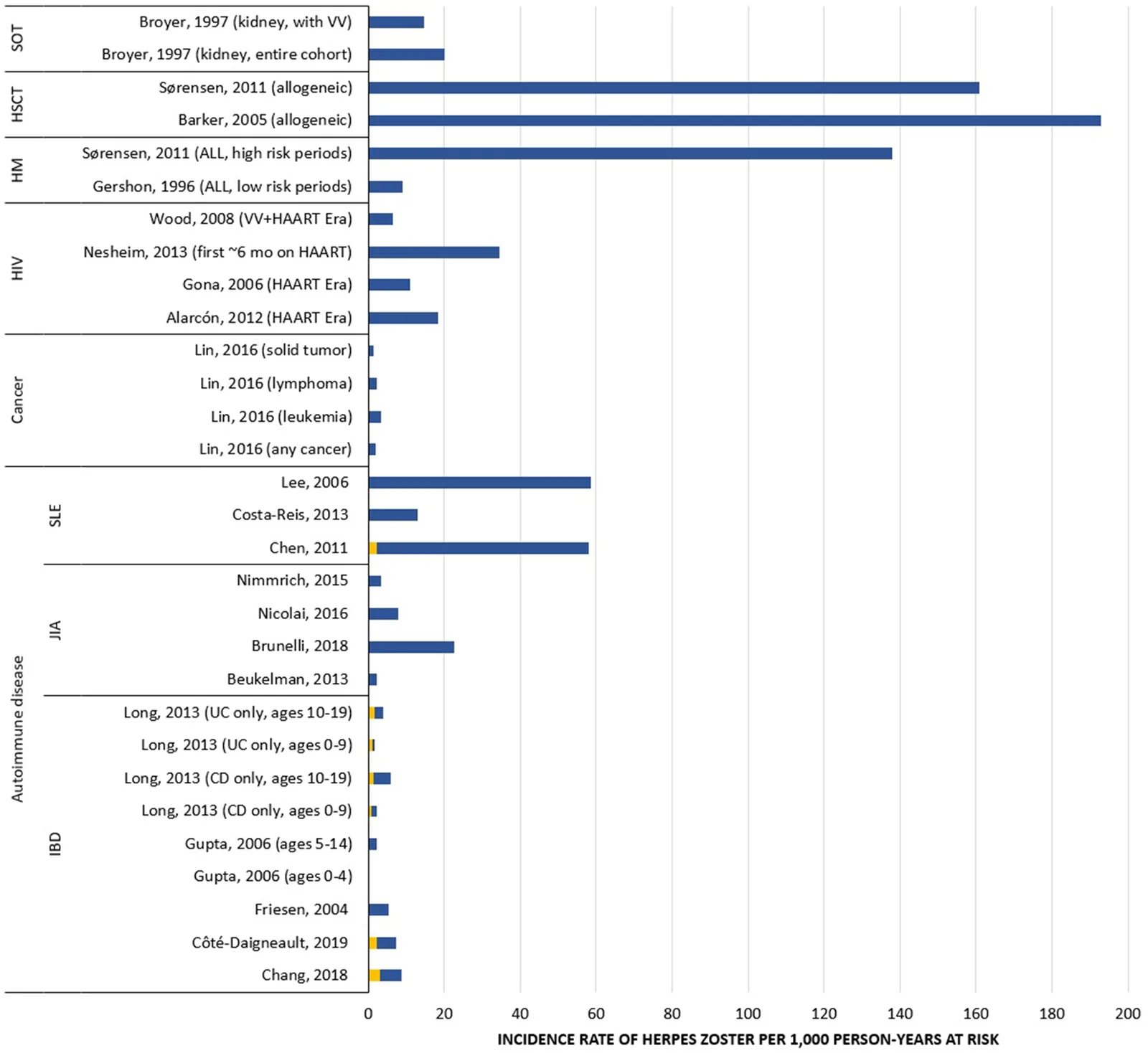
Incidence rate of herpes zoster in pediatric populations, by immunocompromising condition. The lighter shaded, gold sub-bars represent HZ incidence in age-matched controls without the condition (internal to the study, where available).

The 41 studies provided 73 discrete estimates of HZ occurrence in pediatric populations with the IC conditions of interest. Thirty-four papers provided 43 estimates of HZ risk (cumulative incidence), including 21 in HCT, 6 in cancer, 4 in HIV, 3 in SOT, 4 in SLE, 3 in JIA, and 2 in IBD (Figures 2, 3). Twenty-one papers provided 30 estimates of HZ incidence rate in IBD (9 estimates), JIA (4), SLE (3), cancer (6), HIV (4), HCT (2), and SOT (2) (Figure 4). Five of the incidence studies also reported IRs among matched controls without the condition.

Twenty-one studies (51%) reported the proportions of HZ cases that developed specific complications (Table 2). Four studies reported HZ occurrence results stratified by varicella vaccination (VV) status (12–15).

**Table 2:** Studies reporting complications Among pediatric HZ cases with IC conditions.

IC condition	Study	Complications observed/patients with HZ
JIA	Brunelli et al. (22)	PHN (duration NR): 1/8 (13%) Recurrence: 1/8 (13%)
JIA	Nicolai et al. (23)	PHN: 0/3 episodes^a^ DZ: 0/3 episodes^a^ HZO: 0/3 episodes^a^
JIA	Nimmrich and Horneff (24)	PHN (duration NR): 1/17 (6%) Recurrence: 1/17 (6%)
SLE	Ferreira et al. (15)	PHN (duration: >1 mo after lesion resolution): 6/120 (5%) DZ: 0/120 Overlapping bacterial infection: 16/120 (13%) Ophthalmic involvement: 0/120 Death: 0/120 Recurrence: 6/120 (5%)
SLE	Wu et al. (19)	PHN: 0/35 DZ: 0/35 Death: 0/35 Recurrence: 7/35 (20%)
SLE	Lee et al. (20)	PHN: 0/15 DZ: 0/15 Superimposed bacterial skin infections: 9/19 episodes^a^ (47%) Recurrence: 2/15 episodes^a^ (13%)
HCT (allogeneic)	Arici et al. (12)	PHN: NR DZ: 11/58 (19%) Secondary bacterial skin infection: 8/58 (14%) Death: 0/58
HCT (allogeneic *N* = 12, autologous *N* = 1)	Tatebe et al. (55)	PHN: NR DZ: 1/13 (8%) Death: 0/13
HCT (allogeneic)	Düver et al. (14)	PHN: NR DZ: 1/16 (6%)
HCT (autologous)	Kang et al. (32)	PHN: 2/44 (5%) DZ: 10/51 episodes^a^ (20%) Death: 2/44 (5%) (1 encephalitis, 1 ocular infection w/ visceral involvement) Recurrence: 7/44 (16%)
HCT (allogeneic)	Ryan et al. (56)	PHN: NR DZ: 1/6 (17%) Death: 1/6 (17%)
HCT (allogeneic)	Han et al. (33)	PHN (duration >1 mo): 0/25 DZ: 6/25 (24%) Skin infection: 3/25 (12%) Meningitis: 1/25 (4%) Facial palsy: 1/25 (4%)
HCT (autologous, early post-transplant period)	Choi et al. (57)	PHN: NR DZ: 3/14 (21%) DZ with visceral involvement: 0/14
HCT (likely >95% allogeneic)	Aytac et al. (34)	PHN (duration NR): 2/22 (9%) DZ: 0/22 Death: 0/22
HCT (allogeneic or autologous; all ages *N* = 78)	Onozawa et al. (37)	PHN (any duration beyond rash healing), by age: 0–19: 0% 20–29: 28.4% 30–39: 22.1% 40–49: 38.4% 50–59: 48.1% 60+: 44.4% All ages: 27/78 (35%)
HCT (allogeneic, BMT and CBT)	Vandenbosch et al. (61)	PHN: NR Visceral dissemination (in BMT): 1/24 (4%) Visceral dissemination (in CBT): 6/17 (35%) Death: 0/41
HCT (allogeneic or autologous)	Berman et al. (36)	PHN (any duration beyond lesions): 21/73^b^ (29%; 95% CI: 19%, 41%) Disseminated skin involvement: 15/74^b^ (20%; 95% CI: 12%,31%) Visceral organ involvement: 5/75^b^ (7%; 95% CI: 2.2%, 15%) Death: 1/76^b^ (1%) (lung involvement) Relapsed infection^c^: 4/76^b^ (5%) Recurrence: 4/76^b^ (5%)
HCT (allogeneic or autologous)	Leung et al. (35)	PHN (duration: 10 days, resolved): 1/27 (4%) DZ: 13/27 (48%) Visceral dissemination: 3/27 (11%) Death: 0/27 Recurrence: 0/27
HCT (allogeneic or autologous)	Maltezou et al. (62)	PHN: NR Visceral dissemination: 0/17 Secondary bacterial infection: 0/17
HM (acute lymphoblastic leukemia)	Sørensen et al. (31)	PHN (“severe,” > 2 mo in 2, <2 mo in 3): 5/63 (8%) Secondary bacterial skin infection: 2/63 (3%) Cutaneous dissemination: 11/90 episodes^a^ (12%) Visceral dissemination: 0/63 Death: 0/63 Recurrence: 14/63 (22%)
SOT (kidney)	Broyer et al. (13)	PHN: NR 11/69^b^ (16%) had disease that was “severe and of long duration” (“extensive vesicles, fever more than 38 deg C, evolution longer than 15 days, persistent pain”) Relapsed infection: 6/76^b^ (8%)

HZ, herpes zoster; IC, immunocompromising; JIA, juvenile idiopathic arthritis; PHN, postherpetic neuralgia; NR, not reported; DZ, disseminated zoster; HZO, herpes zoster ophthalmicus; SLE, systemic lupus erythematosus; BMT, bone marrow transplant; CBT, cord blood transplant; HM, hematologic malignancy; SOT, solid organ transplant.

a Denominator measured in total HZ episodes rather than number of patients with HZ (i.e., including recurrences), thus providing a proportion of episodes that resulted in complication.

b Denominators differ because medical record review did not always provide sufficient detail to determine whether a given complication occurred.

c Defined as “reappearance of classic lesions within 2 months of the first episode” (p. 74).

### Autoimmune diseases

#### Systemic lupus erythematosus (SLE)

Six studies involving 2,221 pediatric SLE patients were included (15–20). Across the studies, mean follow-up time ranged from one to two years (15) to about eight years (16), with a median of 4.95 years. The mean or median age at the start of follow-up ranged from approximately 11 (20) to 15 (15) across the four studies that provided this information.

Four studies reported HZ risk, ranging from 14.1% (mean follow-up: 1–2 yr) to 35.7% (mean follow-up: 4.9 yr). Three studies reported a wide range of estimates of HZ IR: 13 (17), 58.1 (18) and 58.7 (20) cases per 1,000 PY. Children in the study reporting low IR were in US birth cohorts for whom VV uptake was very high, while children in the other studies were in South Korea and Hong Kong birth cohorts that lacked widespread VV (21). One study reported HZ IR relative to matched non-SLE controls (IRR = 26.4) (18).

Across three studies, PHN lasting over a month developed in 6/170 HZ cases (3.5%). None of the subjects developed disseminated zoster and none died (15, 19, 20).

#### Juvenile idiopathic arthritis (JIA)

Four studies involving 11,808 JIA patients were included (22–25). Three reported HZ risk ranging from 0.4% to 7.5% (median: 0.6%). All four reported HZ IR, ranging from 2.25 to 22.6 cases per 1,000 PY (median: 5.6 per 1,000 PY). Across the four studies, the mean age at the start of follow-up ranged from seven (23) to 15 years (22). The mean duration of follow-up ranged from 1.6 (25) to 3.7 years (22).

Across three studies, PHN of unspecified duration developed in 2/28 HZ episodes (7.1%). Disseminated zoster was not explicitly studied and no other adverse complications were noted (22–24).

#### Inflammatory bowel disease (IBD)

Five studies of pediatric IBD patients were included, four of which were database studies each covering thousands of subjects (Table 1) (26–29, 54). Among three studies that reported IR for similar age groups (0–17, 0–19, and 0–20 year-olds), the IR for HZ ranged from 5.4 to 8.9 episodes per 1,000 PY (median: 7.2) (26–28).

Three studies of IBD – from South Korea, Canada, and the US – all found that pediatric IBD patients experienced HZ at approximately three times the rate of controls (26, 27, 29). The US-based study separated IBD into subtypes, finding a higher relative incidence for Crohn's disease (IRR = 4.6) than for ulcerative colitis (IRR = 2.6) in the 10–19 age group (29).

### Hematopoietic cell transplants (HCT)

Of 18 included studies involving pediatric HCT (12, 14, 30–37, 55–62), malignancies were the dominant underlying condition in most, but non-malignant conditions (most notably *β*-thalassemia, aplastic anemia, and primary immunodeficiencies) were also well-represented (Supplementary Materials). Average follow-up times varied widely (<30 days to 10 years).

Across 17 studies with a median follow-up time of 2.1 years, the median HZ risk was 17.7% (IQR: 10.7%, 31.0%). Two studies reported incidence rates of HZ in HCT recipients with hematologic malignancies (Figure 4); they observed 193 and 161 HZ episodes per 1,000 PY, over average follow-up periods of 0.95 and 2.5 years post-HCT, respectively (30, 31). These were the highest HZ IRs observed in the current review.

**Figure 5:**
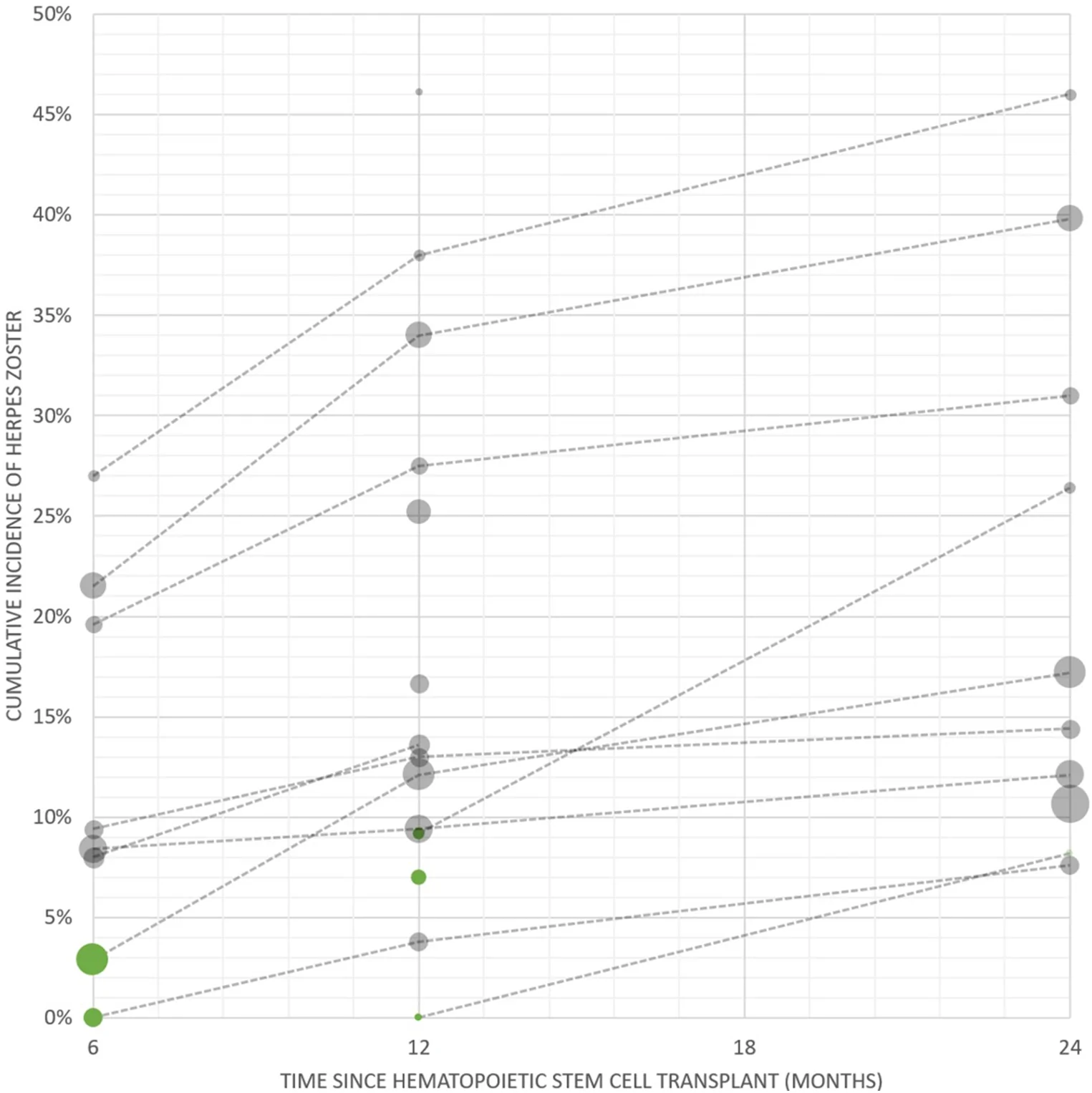
Cumulative incidence of HZ by time since HCT (based on 11 studies). Dashed lines connect the same cohort over time. Green nodes denote that antiviral prophylaxis was used almost continuously in nearly the entire cohort up to that time. Larger nodes denote larger study samples (radius proportionate to square root of sample size). For cohorts with significant mortality during follow-up, it was a requirement that interim risk estimates were produced using a method that treated death as a competing risk. Sources: Arıcı et al. (12), Aytaç et al. (34), Berman et al. (36), Goussetis et al. (58), Han et al. (33), Kang et al. (32), Leung et al. (35), Ryan et al. (56), Tatebe et al. (55), Vandenbosch et al. (61), Vermont et al. (60).

HZ risk over post-HCT time was charted for the 11 studies that reported cumulative incidence of HZ at common (study-interim) time points (Figure 5). Patients who developed HZ within 24 months post-HCT usually did so within the first six months, unless they were in study populations that received nearly universal continuous antiviral prophylaxis for the first six months (Figure 5).

Six studies of HCT patients reported the frequency of PHN among those who developed HZ using a variety of definitions (32–37). Excluding one study that did not provide sample size information (37) and an outlier study that did not specify a required duration of pain after lesion resolution (36), 5/118 HZ cases (4.2%) developed PHN. The proportions of HZ cases developing PHN were similar across these four studies (Table 2).

Disseminated zoster occurred in 66/307 HZ cases (21.5%) across ten studies (median: 19.3%; range 0% to 48.1%). Visceral dissemination affected 15 out of 174 HZ cases (8.6%) across five studies (median: 6.7%; range: 0% to 17.1%). Proportions of subjects with visceral dissemination were similar across these studies (Table 2).

### Cancer

Three studies with different methods and data sources reported HZ risk and incidence rate in three heterogenous groups of pediatric cancer patients (31, 38, 39). One study that followed children during a high-risk period for HZ [from diagnosis with acute lymphoblastic leukemia (ALL) through early treatments; average follow-up: 2.9 years] reported the highest IR (138 HZ episodes per 1,000 PY) and risk [63 of 226 patients (27.9%)] (31). The second study, an open-label trial of VV in 511 VZV-seronegative children with remitted ALL found that 3.1% of subjects developed HZ over approximately the next four years (IR = 9 HZ episodes per 1,000 PY) (38). Although both studies followed patients with the same underlying condition, they involved subjects with different expected HZ risk because of differences in disease stage and treatment intensity, time elapsed since VZV seroconversion, and whether seroconversion arose from natural infection or vaccination.

The third study used a nationwide insurance claims database to estimate the occurrence of HZ in pediatric subjects with any cancer (0.7% over 3.4 years, IR = 2.1 HZ episodes per 1,000 PY), leukemias (1.1%), lymphomas (0.8%), and solid tumors (0.4%) (39). The relatively low HZ frequencies in this study likely reflect a stringent HZ case definition (requiring at least three ambulatory or one inpatient claim). Relative to age- and sex-matched controls without cancer, HZ incidence was significantly elevated [IRR (95% CI) for any cancer: 8.6 (4.8–15.6)] and varied by type of cancer [hazard ratio (95% CI) for leukemia, lymphoma, solid tumors: 13.8 (6.9–27.6), 8.3 (2.8–24.9), 5.3 (2.5–11.3)] (39).

Of the three studies, one – of children with acute lymphoblastic leukemia – reported on HZ complications (31). “Severe” PHN developed in 5/63 patients with HZ (7.9%), of whom three saw PHN resolution within two months. Fourteen patients experienced a total of 27 episodes of HZ recurrence. Eleven of 90 HZ episodes were disseminated (12.2%), but visceral dissemination was not observed (31).

### Solid organ transplant

Two studies reported on the occurrence of HZ after pediatric subjects received kidney transplants, with the risk of HZ 6.7% (mean follow-up: 4.6 yr) in one report (40) and 10.8% (mean follow-up: approx. 5–6 yr) in the other (13). The latter study observed greater risk and IR of HZ among patients who, at the time of transplant, were VZV-seropositive through chickenpox infection (13.0%, 21.7 HZ episodes per 1,000 PY, *n* = 415) than those seropositive through VV (7.1%, 14.7 HZ episodes per 1,000 PY, *n* = 212) (no statistical test performed). The HZ IR among all transplantees was approximately 20 HZ episodes per 1,000 PY (based on information reported for patient subgroups) (13). Additionally, while specific complications were not tabulated, at least 11/76 HZ cases were considered to be severe, defined as having “extensive vesicles, fever more than 38 °C, evolution longer than 15 days, persistent pain” (13).

### HIV

Four studies of HZ among pediatric HIV patients on HAART were identified, three in the US (41–43) and one across multiple Latin American countries but predominantly Brazil (66% of subjects) (44). All were conducted between 2000 and 2007, and all reported HZ risk and IR. HZ risk ranged from 1.3% (over approximately the first six months on HAART) to 6.2% (median follow-up: 3.9 yr), with a median of 2.3%. The median incidence rate of HZ was 14.7 per 1,000 PY, ranging from 6.5 per 1,000 PY among US children in 2000–2006 (age range not reported) (43) to 34.5 per 1,000 PY among 524 US infants, children, and adolescents (ages 0–24 in 2005–2006) with low VV uptake (11.5%) during their first six months of HAART (41). The HZ IR was nominally higher in the Latin American study (18.3 per 1,000 PY) (44) than in the most directly comparable US study (11.1 per 1,000 PY) (42), possibly reflecting the earlier adoption of VV in the US.

### Modification of HZ risk by varicella vaccination

The four studies that reported HZ occurrence in relation to VV status all found that subjects with a history of VV were less likely to develop HZ than subjects without VV (12–15). Pooling across the four studies – which involved subjects with SLE, HCT, and kidney transplantation – 20 out of 279 (7.2%) of subjects with a history of VV developed HZ, compared to 239 out of 1,555 (15.4%) of subjects without a history of VV (pooled risk ratio: 0.47). Study-specific data related to VV is presented in Supplementary Table 3.

## Discussion

This review organizes and distills available literature on the burden of HZ in children with a variety of IC conditions. HCT, leukemia treatment generally, and SLE stood out as conditions associated with a particularly high risk of HZ. The incidence of HZ in IC children compared to healthy pediatric controls differed by IC condition, with incidence rate ratios ranging from considerably elevated (∼3 in IBD) to highly elevated (26.4 in SLE, 14.2 in leukemia – and higher for certain periods of treatment such as HCT). Moreover, children with a range of IC conditions were at high risk of developing acute complications of HZ such as secondary bacterial infections and disseminated zoster. Disseminated zoster of any kind post-HCT was common [median across 10 estimates of 19.3% (IQR: 9.9%, 23.4%)] and exceeded even the proportion previously reported in adults undergoing HCT [3% (IQR: 0.2%, 3.8%)] (11). Visceral dissemination, a serious complication of HZ, affected a striking 15/174 HZ cases (8.6%) after HCT across five studies. Together, these findings indicate that both HZ incidence and disease severity are substantially increased among children with certain immunocompromising conditions, particularly those undergoing HCT.

In children undergoing HCT, the risk of PHN was clearly elevated compared to that of children in the general population, and elevated PHN risk was also suggested among children with SLE and JIA. However, across all conditions studied, PHN was broadly less likely to develop in children than in adults with the same IC conditions (cf. Chen et al., 2014; McKay et al., 2020). This finding is consistent with results of a large hospital-based surveillance study in Canada (not included in the current review because it analyzed all IC conditions grouped together), which found that physician-diagnosed PHN developed in 4/577 (0.7%) hospitalized children with IC conditions (45). That same study also showed elevated proportions of disseminated zoster (10.1%) and secondary infection (10.4%), which are consistent with our findings.

Interpretation of the findings of this review should also consider the changing epidemiology of pediatric HZ following the introduction of routine childhood varicella vaccination (VV). Early childhood VV appears to substantially reduce the risk of pediatric HZ in the general population (10, 46), yet only four included studies in our review reported individual-level VV status. Moreover, most studies in this review were conducted among children born before widespread adoption of routine childhood VV. The median study period began in 2000 and ended in 2007, and the median age of participants at the start of follow-up was at least five years; by comparison, the first country to achieve widespread VV uptake (the United States) did so around the year 2002 (47). Consequently, the HZ incidence and complication rates presented in this review may overestimate the current burden of disease among pediatric IC populations in countries that subsequently achieved high VV coverage. At the same time, however, these findings remain highly relevant for much of the world. Data from the World Health Organization indicate that as of 2025, 133 out of 194 countries (69%) had not introduced VV into their national immunization programs, and another 10 (5%) had done so only partially or for select risk groups (48). Even among countries with the highest levels of economic development, 18 out of 40 (45%) lacked a national VV immunization program. More research is therefore needed to characterize HZ risk among pediatric IC populations across settings with different levels of VV uptake. This need is underscored by the limited availability of individual-level VV data in the existing literature, which hindered direct comparisons across studies.

Although limited, the available evidence from this review supports a protective effect of VV against HZ that has previously been observed in the general pediatric population (10, 46). All four studies that reported results stratified by VV status found lower HZ risk among vaccinated than unvaccinated children with IC conditions (Supplementary Table 3) (12–15). In addition, a fifth study of HIV-positive children in the US, which did not report individual VV status, observed lower HZ incidence in the post-VV era (2000–2006) than during earlier periods, independent of the introduction of HAART (43). Our findings are consistent with those of a case-control study of pediatric leukemia patients predating our review period that found HZ incidence in patients receiving VV was approximately one-third of that in matched patients who became seropositive for VZV through natural infection (chickenpox) (49). Although evidence remains sparse, understanding the relationship between VV and HZ risk in immunocompromised children is particularly important because VV can be safely given after diagnosis to select patients with IC conditions.

The studies informing the HZ frequency estimates in this review used either medical records or claims data as primary data sources. The use of ICD code-based HZ case definitions has been validated against medical records and been found to be highly sensitive with positive predictive value (PPV), ranging from 85% to 97% (50–53). Three validation studies included pediatric subjects, two of which reported age-stratified results, showing similar PPV in pediatric subjects as in adults (50, 52). By contrast, HZ complications such as PHN have been shown to be poorly captured in administrative data, leading to underestimation of their frequency (9). The results pertaining to HZ complications in this review (Table 2) were derived exclusively from medical record-based studies.

Our review has some additional limitations. First, as noted earlier, VV is known to substantially reduce HZ risk in general pediatric populations, but most studies reviewed lacked information on individual VV history, which prevented stratification or control for this exposure. Where possible, we reported HZ risk stratified by VV status, including relative risk comparing vaccinated and unvaccinated subjects. Lack of VV data also prevented analysis of HZ complications in relation to VV status. More generally, differences in study settings, patient management, follow-up duration, and underlying immunocompromising conditions limited direct comparisons across studies and precluded formal meta-analysis. Variation in immunosuppressive treatment regimens both within and between conditions is also likely to have contributed substantially to differences in observed HZ incidence and complications. Additional study details provided in the Supplementary Materials, may assist readers in interpreting individual study results.

The studies collected in this review may not be exhaustive of the relevant literature. Although databases beyond PubMed may have identified additional publications, we sought to minimize this risk by screening the reference lists of all 159 full-text reports reviewed for eligibility. Even so, relevant studies indexed in other databases may have been missed. Similarly, restricting the review to articles published in English may have resulted in the exclusion of relevant literature, and may have limited the geographic diversity of the evidence base to higher-income countries with more established VV programs. Nevertheless, the included literature was drawn from 22 distinct countries and territories spanning North and South America, the Caribbean, Europe, and Asia. While these methodological decisions may have affected the completeness of the literature identified, they are unlikely to have systematically affected the representativeness of the evidence identified. Finally, although the set of IC conditions considered in this review (HCT, cancer, SOT, HIV on HAART, or any autoimmune disease) provides broad coverage of immunocompromised pediatric populations, some less commonly diagnosed but clinically important IC conditions such as primary immunodeficiencies were outside the scope of this review and remain important areas for investigation.

To our knowledge, this is the first review to comprehensively synthesize published data on the risk of herpes zoster among immunocompromised children. Many of the included studies were conducted in pediatric specialty settings, facilitating complete case ascertainment and accurate reporting of complications, such as PHN. These findings provide clinicians with a consolidated overview of the populations at greatest risk for HZ and its complications, which may help inform clinical vigilance, patient counseling, and preventive strategies. Despite the available evidence, important knowledge gaps remain. Future research should prioritize prospective, multicenter studies using standardized definitions and reporting of pediatric herpes zoster outcomes to improve comparability across studies and strengthen estimates of disease burden. Additional investigation is needed in relatively understudied but increasingly prevalent pediatric conditions, such as inflammatory bowel disease, as well as in children with primary immunodeficiencies, who may be at particularly high risk of both primary varicella infection and HZ. Finally, further studies are warranted to evaluate the extent and durability of VV–derived protection against severe HZ in immunocompromised children and to determine how this protection varies across underlying conditions. As routine childhood VV continues to expand worldwide, the epidemiology of pediatric HZ is likely to evolve, underscoring the importance of continued surveillance and periodic reassessment of disease burden over time.
