# Supplementary material for: Herpes zoster frequency and severity in immunocompromised pediatric populations: a structured narrative review
Source: Front Pediatr. 2026 Aug 28;14:1900439. doi: 10.3389/fped.2026.1900439 (PMC13563003; doi:10.3389/fped.2026.1900439)
Supplement: Supplementary file 1 [file Supplementaryfile1.docx]

## Supplement to “Herpes Zoster Frequency and Severity in Immunocompromised Pediatric Populations”

### Details of Database Search Strategy

The search strategy was based on that of McKay et al. 2020 and adapted to identify pediatric subjects, include autoimmune diseases, and not exclude any countries.

**Search Part I** followed McKay et al. 2020 except for the underlined portions.

**Search Part I**

(Herpes Zoster[mesh] OR “Neuralgia, Postherpetic”[mesh] OR shingles OR “postherpetic neuralgia”)

AND (“inciden*”[tiab] OR “prevalen*”[tiab] OR “epidemiology”[subheading] OR “epidemiol*”[tiab])

AND (Hematologic diseases[mesh] OR neoplasm[mesh] OR "suppress immune" OR Hematopoietic Stem Cell Transplantation[mesh] OR Immunocompromised host[mesh] OR immune system diseases[mesh] OR Immune System Phenomena[mesh] OR "altered immune function" OR immunocompr* OR immunosup* OR immunodef* OR "HIV infections"[mesh] OR "Transplant Recipients"[Mesh] OR "inflammatory bowel diseases"[mesh] OR Psoriasis[mesh] OR "Immune Tolerance"[mesh] OR "vitamin D"[mesh] OR "Disease Susceptibility"[mesh] OR “Hematologic diseases” OR neoplasm OR “Hematopoietic Stem Cell Transplantation” OR “Immunocompromised host” OR diabetes OR “immune system diseases” OR “Immune System Phenomena” OR “bone marrow transplant” OR “stem cell transplant” OR HIV OR “transplant recipients” OR “inflammatory bowel diseases” OR IBD OR Psoriasis OR “Immune Tolerance” OR “Disease Susceptibility” OR “opportunistic infections” OR cancer OR chemotherapy OR radiation OR radiotherapy OR Hodgkin's OR Non-Hodgkin's OR Leukemia OR Myeloma OR “Immune Disorder” OR “immunity deficiency” OR “Congenital hypogammaglobulinemia” OR “immunoglobulin deficiencies” OR Macroglobulinemia OR “kidney transplant” OR “renal transplant” OR glomerul* OR malignancy OR AIDS OR “human immunodeficiency virus” OR asthma OR asthma[mesh] OR “oral corticosteroids” OR lupus OR autoimmun*)

AND (journal article[pt] OR review[pt] OR clinical study[pt] OR "Meta-Analysis"[pt] OR "Multicenter Study" [pt] OR "Comparative Study"[pt] OR "Clinical Trials as Topic"[Mesh] OR "Research Support, Non-U.S. Gov't"[pt] OR "Research Support, U.S. Government" [pt] OR Randomized Controlled Trial[pt])

AND (Humans[Mesh])

AND ("1993"[Date - Publication] : "3000"[Date - Publication])

AND (english[la])

NOT ("Clinical Laboratory Techniques"[Mesh])

NOT (letter[pt] OR editorial[pt])

The above search, performed on 9/15/2024, yielded 2,036 results. After restriction by the PubMed age filter “Child: birth-18 years,” 538 results remained. When also including “young adult: 19-24,” the search pulled 616 results.

**Search Part II** was identical to **Search Part I** except for the addition of “AND (“pediatric”[tiab] OR “child*”[tiab] OR “infant*”[tiab])” after the second line. No age filters were used. This search, also performed on 9/15/2024, yielded 267 results.

There were 207 duplicates in the union of the two searches, meaning the second search contributed an additional 60 unique records beyond the first search, for a total of 676 records that underwent abstract/title review.

Since heterogeneity of study populations and sparseness of literature for particular IC conditions or HZ outcomes were expected, validation studies assessing the performance of different methods to restrict database searches by age guided the choice of the PubMed age filters and keywords used to target pediatric subjects (Kastner et al., 2006; Leclercq et al., 2013).

### Additional Notes Related to Table 1

Three studies of HSCT (Arıcı et al., 2024; Choi et al., 2016; Maltezou et al., 2000) reported results in terms of transplants, not patients. Sample size for these studies therefore refers to number of transplants. For two of the studies, repeated transplants were uncommon. In the third study (Choi et al., 2016), transplants were part of planned tandem autologous HSCT. 324 patients underwent the first transplant, of whom 283 underwent a second transplant.

Two studies of HSCT (Blennow et al., 2014; Vandenbosch et al., 2008) were restricted to subjects seropositive for VZV. A third (Wood et al., 2008) was restricted to subjects with a documented history of primary varicella infection.

The following values in the table were estimated based on close reading of the texts and relied on assumptions that were considered extremely likely to obtain: median age at start of follow-up (Aytaç et al., 2011; Ferreira et al., 2016; Vandenbosch et al., 2008; Vermont et al., 2014); mean age at start of follow-up (Gershon et al., 1996); interquartile range of ages at start of follow-up (Gona et al., 2006); follow-up time per subject, non-cases only (Leung et al., 2000), follow-up time per subject (Gershon et al., 1996).

One study of SLE (Yu et al., 2022) did not report the number of subjects who experienced HZ (n), so HZ risk (n / N) could not be calculated. The study used the Kaplan-Meier method to estimate the cumulative incidence. Because mortality and loss to follow-up were low, this estimate of cumulative incidence was considered comparable to n / N, and this study was included.

### Supplemental Table 1. Additional Study Details

| **Study** | **Data Source \|\| Source Population \|\| Important Aspects of Subject Selection or HZ Case Definition** |
| --- | --- |
| Côté-Daigneault, 2019 | Administrative healthcare database \|\| nearly the entire population of Quebec, Canada \|\| 4 IBD physician billings in a 2-year period, or 1 IBD hospitalization |
| Chang, 2018 | Insurance claims database and registry for rare and intractable diseases \|\| practically the entire population of South Korea \|\| IBD-specific prescription medication; diagnosis of exactly one of CD or UC |
| Long, 2013 | Insurance claims database \|\| Approximately 60 million people in the US under age 65 with commercial health insurance |
| Gupta, 2006 | Database of primary care medical records \|\| ~6% population-based sample of the UK \|\| diagnosis of exactly one of CD or UC |
| Friesen, 2004 | Infliximab infusion database, hospital pharmacy records and patient charts \|\| all IBD patients starting infliximab at pediatric gastroenterology section of a children’s hospital in Kansas City, MO, US |
| Brunelli, 2018 | Electronic medical records \|\| biologic therapy center of rheumatology division in a São Paulo, Brazil hospital \|\| patients followed from start on any of five biologics; must have completed at least 8 weeks of therapy |
| Nicolai, 2016 | “Patient charts and/or parents and patient interview” \|\| rheumatology division of a children’s hospital in Rome, Italy \|\| patients consecutively evaluated in 2010 and receiving either etanercept and methotrexate (N=85) or methotrexate alone (N=71) |
| Nimmrich, 2015 | Pediatric biologics registry \|\| patients at multiple clinical sites in Germany participating in an open-label study of biologics \|\| patients followed from start of biologic or methotrexate |
| Beukelman, 2013 | Medicaid medical and pharmacy claims data \|\| children from lower-income families in all 50 US states \|\| 2 JIA physician diagnosis codes or 1 with JIA-specific prescription |
| Yu, 2022 | Insurance claims database and a registry for catastrophic illnesses \|\| practically the entire population of Taiwan \|\| individuals with a new SLE diagnosis in 2005 were followed until first HZ episode, death, or the end of 2013 |
| Ferreira, 2016 | Medical records \|\| new and existing SLE patients (onset before age 18) at the ten pediatric rheumatology tertiary referral services of São Paulo state, Brazil |
| Costa-Reis, 2013 | Medical charts \|\| pediatric rheumatology division of a New York City hospital \|\| 3+ clinic visits during 2007-2009; follow-up started at diagnosis or first encounter |
| Chen, 2011 | Insurance claims database and a registry for catastrophic illnesses \|\| practically the entire population of Taiwan \|\| new SLE diagnosis (or resumption of health care utilization after 2+ year hiatus) and 3 years follow-up available after such time |
| Wu, 2011 | Clinical data \|\| pediatric-onset SLE patients under regular follow-up at a hospital in Taiwan |
| Lee, 2006 | Medical records \|\| pediatric immunology clinic in a Hong Kong hospital |
| Arici, 2024 | Medical records and phone interviews with families \|\| first or second allogeneic HSCTs performed at a children’s hosptal in Turkey \|\| common underlying conditions: thalassemia major (39.9%), acute leukemia (28.5%) |
| Tatebe, 2022 | Medical records \|\| first-time HSCT recipients at a hospital in Japan, except patients with less than three months of follow-up (7 out of 60 otherwise eligible patients) \|\| common underlying conditions: hematological (47.2%), solid (34.0%) malignancies; second HSCT ended the follow-up |
| Düver, 2020 | Medical records \|\| first-time allogeneic HSCT recipients at a children’s hospital in Germany \|\| common underlying conditions: hematological (57.0%) or other (22.4%) malignancies; second HSCT ended the follow-up |
| Kang, 2020 | Medical records \|\| first or second autologous HSCT recipients at a pediatric cancer center in South Korea \|\| underlying conditions were malignancies: neuroblastoma (41.4%), brain tumor (35.1%) |
| Ryan, 2019 | Medical records \|\| first-time allogeneic HSCT recipients at a children’s hospital in Australia \|\| common underlying conditions: malignancies (60.6%, large majority hematological), non-malignant diseases (39.4%, including 10 primary immunodeficiencies) |
| Han, 2017 | Medical records \|\| allogeneic HSCT recipients at a South Korea academic hospital \|\| common underlying conditions: severe aplastic anemia (30.0%), acute lymphoblastic leukemia (28.1%), acute myeloid leukemia (24.4%), other malignancies (12.4%), other non-malignancies (5.1%) |
| Choi, 2016 | Medical records \|\| all transplants in planned tandem autologous HSCTs in pediatric patients with high-risk or recurrent solid tumors performed at a single center |
| Goussetis, 2015 | Medical records \|\| patients with hemoglobinopathies (95% β-thalassemia) who received allogeneic HSCT at a children’s hospital in Greece |
| Blennow, 2014 | Medical records \|\| allogeneic HSCT recipients at a university hospital in Sweden \|\| restricted to VZV-seropositive subjects |
| Vermont, 2014 | Medical records \|\| first-time allogeneic HSCT recipients in the pediatric department of a hospital in the Netherlands \|\| common underlying conditions: acute leukemia (43.6%), primary immunodeficiencies (17.2%), other hematologic malignancies (12.9%), hemoglobinopathies (11.0%) |
| Aytac, 2011 | Medical records \|\| HSCT recipients in the pediatric department of a hospital in Turkey, except patients who died within three months of HSCT (number NR) \|\| both autologous and allogeneic HSCTs were eligible, but allogeneic transplants predominated (likely >95%) |
| Onozawa, 2009 | Medical records \|\| all patients undergoing either allogeneic or autologous HSCTs at any of 19 centers in Hokkaido prefecture, Japan |
| Vandenbosch, 2008 | HSCT research database and patient medical charts \|\| first-time allogeneic HSCT recipients in a pediatric unit of a hospital in Montreal, Canada, except patients seronegative for VZV (19 out of 133 otherwise eligible patients) \|\| second HSCT ended the follow-up |
| Berman, 2006 | HSCT research database and patient medical charts \|\| HSCT recipients at a children’s hospital in Boston, MA, US |
| Barker, 2005 | Medical records \|\| all pediatric (0-18 y/o) patients undergoing a first allogeneic HSCT for HM at the University of Minnesota |
| Leung, 2000 | Medical records \|\| HSCT recipients in the pediatric department of a hospital in Hong Kong |
| Maltezou, 2000 | Medical records \|\| HSCTs performed in the pediatrics division of M. D. Anderson Cancer Center in Houston, TX, US \|\| subjects followed to 2 years post-HSCT |
| Gershon, 1996 | Open-label trial of VV \|\| VZV-seronegative children with acute lymphoblastic leukemia in remission for at least year who then received VV (at start of follow-up) |
| Sørensen, 2011 | Medical records \|\| nearly all (97%) of the children diagnosed and treated for acute lymphoblastic leukemia at two hospitals in Denmark \|\| some results restricted to the 160 out of 226 patients with laboratory evidence of VZV seropositivity at diagnosis |
| Lin, 2016 | Insurance claims database and a registry for catastrophic illnesses \|\| practically the entire population of Taiwan \|\| newly diagnosed cancer patients; HZ case definition required 3 ambulatory claims or 1 inpatient claim |
| Chavers, 1997 | Medical records and outpatient transplant charts \|\| All patients <18 y/o who underwent primary renal transplant at the University of Minnesota during the study period |
| Broyer, 1997 | Medical records \|\| children who underwent renal transplant at a children’s hospital in Paris, France, except the 56 of 760 of transplantees (7.4%) who died or lost their transplant within three months |
| Nesheim, 2013 | Medical records \|\| HIV-infected infants, children, and adolescents (ages 0-24) receiving care at one of 22 HIV specialty clinics across the US and participating in a prospective cohort study \|\| participation rate in the larger prospective cohort study was high (83%); among participants, complete medical record abstraction was low (28%) but not obviously inducing bias |
| Alarcón, 2012 | Medical data \|\| childen vertically-infected with HIV receiving care at one of 17 clinics in Latin America (subjects from Brazil (66.1%), Mexico (16.1%), Argentina (9.7%), Peru (5.1%), Jamaica (3.0%)) |
| Wood, 2008 | Clinical database and directed chart review \|\| children perinatally infected with HIV receiving care at the HIV Clinic of the Children’s Hospital of Philadelphia \|\| restricted to subjects with a documented history of primary varicella infection |
| Gona, 2006 | Medical data \|\| HIV-infected children in the US and Puerto Rico |

### Supplemental Table 2. Additional Selected Findings

| **Study (IC Condition)** | **Additional Selected Findings** |
| --- | --- |
| Gupta, 2006 (IBD) | Age \| HZ IR per 1,000 PY; 0-4 \| 0; 5-14 \| 2.37; 15-44 (for comparison) \| 3.43 |
| Yu, 2022 (SLE) | Time since follow-up began (y) \| HZ risk (cumulative incidence); About 8.4 (entire study time) \| 21.3%; 1 \| 8.9%; 2 \| 12.4%; 5 \| 18.7% |
| Blennow, 2014 (HSCT) | Age \| HZ risk (cumulative incidence); 0-9 \| 24%; 10-19 \| 40%; 20-29 \| 17%; 30-39 \| 24%; 40-49 \| 15%; 50+ \| 18% Risk factors for VZV reactivation in a multivariate regression analysis included age 0 to 19 years (relative hazard: 1.68, 95% CI: 1.21, 2.35) |
| Vermont, 2014 (HSCT) | Antiviral prophylaxis \| HZ risk (cumulative incidence); Received in first 100 days \| 0 / 47 = 0%; Did not receive in first 100 days \| 41 / 116 = 35.3% |
| Sørensen, 2011 (HM including potential HSCT) | Age \| Treatment phase \| Avg. follow-up (y) \| HZ risk \| HZ IR; 1-14 \| All phases \| 2.9 \| 63 / 226 = 27.9% \| 138; 1-5 \| Primary chemo and immune recovery \| 2.5 \| 21 / 85 = 24.7% \| 130; 6-14 \| Primary chemo and immune recovery \| 2.3 \| 26 / 75 = 34.7% \| 200; 1-14 \| HSCT \| 2.5 \| 11 / 30 = 36.7% \| 161; 1-14 \| First three years post-relapse (excluding HSCT) \| 1.2 \| 7 / 29 = 24.1% \| 360 |
| Wood, 2008 (HIV) | Era \| HZ IR (95% CI); All study eras (1989-2006) \| 17.4 (11.1, 25.8); Pre-VV, Pre-HAART era (1989-1996) \| 30.0 (14.4, 55.2); Pre-VV, Post-HAART era (1997-1999) \| 31.9 (14.6, 60.6); VV+HAART era (2000-2006) \| 6.5 (2.1, 15.2) |

### Supplemental Table 3. Studies Reporting HZ Risk Stratified by VV Status

| **Study (IC Condition)** | **VV+**  **(HZ / N)** | **VV+**  **HZ Risk** | **VV-**  **(HZ / N)** | **VV-**  **HZ Risk** | **HZ Risk Ratio**  **(VV+ to VV-)** |
| --- | --- | --- | --- | --- | --- |
| Ferreira, 2016 (SLE) | 3 / 24 | 12.50% | 117 / 828 | 14.13% | 0.88 |
| Arici, 2024 (HSCT) | 2 / 33 | 6.06% | 56 / 240 | 23.33% | 0.26 |
| Duver, 2020 (HSCT) | 0 / 10 | 0% | 5 / 23 | 21.74% | 0 |
| Broyer, 1997 (SOT) | 15 / 212 | 7.08% | 61 / 464 | 13.15% | 0.54 |
| Pooled Totals | 20 / 279 | 7.17% | 239 / 1555 | 15.37% | 0.47 |

References

ARıCı, G., INCE, E., INCE, E., ILERI, T., CIFTCI, E., DOGU, F., OZDEMIR, H., CAKMAKLI, H. F. & ERTEM, M. 2024. Varicella-Zoster Virus Reactivation After Pediatric Allogeneic Hematopoietic Stem Cell Transplantation, Single-Center Experience of Acyclovir Prophylaxis. *Pediatr Transplant,* 28**,** e14819.

AYTAÇ, S., YALÇIN, S. S., KÜÇÜKBAYRAK, O., ÇETıN, M. & UÇKAN, D. 2011. Dynamics in children and adolescents who experience varicella zoster virus infections after haematopoietic stem cell transplantation: a case-control study. *Epidemiol Infect,* 139**,** 1701-9.

BLENNOW, O., FJAERTOFT, G., WINIARSKI, J., LJUNGMAN, P., MATTSSON, J. & REMBERGER, M. 2014. Varicella-zoster reactivation after allogeneic stem cell transplantation without routine prophylaxis--the incidence remains high. *Biol Blood Marrow Transplant,* 20**,** 1646-9.

CHOI, Y. B., YI, E. S., KANG, J. M., LEE, J. W., YOO, K. H., KIM, Y. J., SUNG, K. W. & KOO, H. H. 2016. Infectious Complications during Tandem High-Dose Chemotherapy and Autologous Stem Cell Transplantation for Children with High-Risk or Recurrent Solid Tumors. *PLoS One,* 11**,** e0162178.

FERREIRA, J. C., MARQUES, H. H., FERRIANI, M. P., GORMEZANO, N. W., TERRERI, M. T., PEREIRA, R. M., MAGALHÃES, C. S., CAMPOS, L. M., BUGNI, V., OKUDA, E. M., MARINI, R., PILEGGI, G. S., BARBOSA, C. M., BONFÁ, E. & SILVA, C. A. 2016. Herpes zoster infection in childhood-onset systemic lupus erythematosus patients: a large multicenter study. *Lupus,* 25**,** 754-9.

GERSHON, A. A., LARUSSA, P., STEINBERG, S., MERVISH, N., LO, S. H. & MEIER, P. 1996. The protective effect of immunologic boosting against zoster: an analysis in leukemic children who were vaccinated against chickenpox. *J Infect Dis,* 173**,** 450-3.

GONA, P., VAN DYKE, R. B., WILLIAMS, P. L., DANKNER, W. M., CHERNOFF, M. C., NACHMAN, S. A. & SEAGE, G. R., 3RD 2006. Incidence of opportunistic and other infections in HIV-infected children in the HAART era. *Jama,* 296**,** 292-300.

KASTNER, M., WILCZYNSKI, N. L., WALKER-DILKS, C., MCKIBBON, K. A. & HAYNES, B. 2006. Age-specific search strategies for Medline. *J Med Internet Res,* 8**,** e25.

LECLERCQ, E., LEEFLANG, M. M., VAN DALEN, E. C. & KREMER, L. C. 2013. Validation of search filters for identifying pediatric studies in PubMed. *J Pediatr,* 162**,** 629-634.e2.

LEUNG, T. F., CHIK, K. W., LI, C. K., LAI, H., SHING, M. M., CHAN, P. K., LEE, V. & YUEN, P. M. 2000. Incidence, risk factors and outcome of varicella-zoster virus infection in children after haematopoietic stem cell transplantation. *Bone Marrow Transplant,* 25**,** 167-72.

MALTEZOU, H. C., KAFETZIS, D. A., ABISAID, D., MANTZOURANIS, E. C., CHAN, K. W. & ROLSTON, K. V. 2000. Viral infections in children undergoing hematopoietic stem cell transplant. *Pediatr Infect Dis J,* 19**,** 307-12.

MCKAY, S. L., GUO, A., PERGAM, S. A. & DOOLING, K. 2020. Herpes Zoster Risk in Immunocompromised Adults in the United States: A Systematic Review. *Clin Infect Dis,* 71**,** e125-e134.

ROHATGI, A. 2022. WebPlotDigitizer. 4.6 ed. https://automeris.io/WebPlotDigitizer.

VANDENBOSCH, K., OVETCHKINE, P., CHAMPAGNE, M. A., HADDAD, E., ALEXANDROV, L. & DUVAL, M. 2008. Varicella-zoster virus disease is more frequent after cord blood than after bone marrow transplantation. *Biol Blood Marrow Transplant,* 14**,** 867-71.

VERMONT, C. L., JOL-VAN DER ZIJDE, E. C., HISSINK MULLER, P., BALL, L. M., BREDIUS, R. G., VOSSEN, A. C. & LANKESTER, A. C. 2014. Varicella zoster reactivation after hematopoietic stem cell transplant in children is strongly correlated with leukemia treatment and suppression of host T-lymphocyte immunity. *Transpl Infect Dis,* 16**,** 188-94.

WOOD, S. M., SHAH, S. S., STEENHOFF, A. P. & RUTSTEIN, R. M. 2008. Primary varicella and herpes zoster among HIV-infected children from 1989 to 2006. *Pediatrics,* 121**,** e150-6.

YU, C. Y., KUO, C. F., CHOU, I. J., CHEN, J. S., LU, H. Y., WU, C. Y., CHEN, L. C., HUANG, J. L. & YEH, K. W. 2022. Comorbidities of systemic lupus erythematosus prior to and following diagnosis in different age-at-onset groups. *Lupus,* 31**,** 963-973.
